# Conformational transition pathway of R308K mutant glucokinase in the presence of the glucokinase activator YNKGKA4

**DOI:** 10.1002/2211-5463.12255

**Published:** 2018-07-06

**Authors:** Nanda Kumar Yellapu, Kalpana Kandlapalli, Ramesh Kandimalla, Pradeepkiran Jangampalli Adi

**Affiliations:** ^1^ Biomedical Informatics Centre Vector Control Research Centre Indian Council of Medical Research Puducherry India; ^2^ Department of Biochemistry Sri Krishnadevaraya University Anantapuramu Andhrapradesh India; ^3^ Garrison Institute on Aging Texas Tech University of Health Science Centre Lubbock TX USA

**Keywords:** glucokinase, molecular dynamics, mutations

## Abstract

Glucokinase (GK) plays a vital role in the control of blood glucose levels and its altered activity can lead to the development of forms of diabetes. We have previously identified a mutant GK (R308K) in patients with type 2 diabetes with reduced enzyme activity. In the present study, the activation mechanism of GK from super‐open to the closed state under wild‐type and mutant conditions in the presence of the novel aminophosphonate derivative YNKGKA4 (an allosteric activator of GK) was characterized via a series of molecular dynamics simulations. A reliable conformational transition pathway of GK was observed from super‐open to closed state during trajectory analysis. Glucose was also observed to modulate its binding orientation in the active site but with stable moments in the cavity. These observations provide insights into the complicated conformational transitions in the presence of YNKGKA4 and the molecular mechanism of GK activators for the allosteric regulation of mutant forms of GK.

**Abbreviations**
GK: glucokinase
MD: molecular dynamics
MODY2: maturity onset diabetes of the young 2
MOE: molecular operating environment
T2D: type 2 diabetes

Glucokinase (GK) is a vital enzyme that plays major role in the control of blood glucose levels both in the liver and pancreatic cells 1. The altered activities of the enzyme lead to pathogenic conditions such as type 2 diabetes (T2D) or maturity onset diabetes of the young 2 (MODY2). The altered activity of the enzyme occurs for several reasons and the major one of these is MODY2 mutations 2. Such genetic modulations in the gene for GK are known to be significant and to cause variation in GK structure and function. The mutations in GK have been observed in patients with hyperglycemia who demonstrate autosomal dominant inheritance and impaired glucose tolerance. Several mutations in the gene for GK have been reported so far that contribute to a hypoglycemic condition in the patients with T2D 3, 4, 5. These mutations are responsible for the altered catalysis of GK, such as increase in glucose *S*_0.5_, a decrease in *V*_max_, and a change in the ATP requirements that affect the *K*_m_ and also the turnover number (*K*_cat_) 6, 7, 8.

Catalysis takes place in the multistep process where, initially, the super‐open state allows the entry of glucose, magnesium and ATP into the active site cavity, followed by the closed state. Next, the super‐open state releases the phosphorylated glucose, enabling entry of the next glucose molecule for catalysis 9. This multistep transition pathway is disturbed by the conformational variations resulting from mutations in the gene for GK. GK activators (GKAs) emerged as a result of the concept that GK with its allosteric activator site can be switched on by any activator molecule 1, 10, 11. GKAs can bind to the allosteric site of GK and enhance its catalytic activity via conformational transitions 12, 13, 14. GKAs regulate the super‐open and closed conformational transitions of GK, thereby promoting its activity, thus leading to utilization of excess blood glucose levels.

In our earlier studies, we reported mutations in GK in a population of T2D patients that are responsible for the hypoglycemic condition 5. Among these mutations, we characterized the R308K mutation *in vitro* and found that GK enzymatic activity was reduced in a mutant condition compared to wild‐type 15. Subsequent to these investigations, a series of GKAs were developed and tested against purified GK from human liver 14. Among the proposed GKAs, ethyl 2‐[3‐[diethoxyphosphoryl‐[[5‐fluoro‐1‐[2‐(hydroxymethyl)‐1,3‐oxathiolan‐5‐yl]‐2‐oxopyrimidin‐4‐yl]amino]methyl]‐4‐oxocyclohexa‐2,5‐dien‐1‐ylidene]‐4‐methyl‐^3^H‐1,3‐thiazole‐5‐carboxylate (PubChem ID: YNKGKA4; Fig. 1) was found to potentially increase GK activity. Accordingly, in the present study, we report the conformational transition of GK from super‐open to closed states in the presence of YNKGKA4, both in wild‐type and R308K mutant conditions. We have observed a confined conformational ensemble in the super‐open state followed by the consistent conformational transition pathway of GK towards the closed state. The overall conformational transitions provide clear insights into how the super‐open state was transformed into the closed state, where the glucose molecule was observed to sit in the binding site cavity, altering its orientation, but not to leave the site. The activator molecule YNKGKA4 was also observed to bind at the allosteric site in a stable form throughout the simulation period, thereby regulating the transition pathway of GK. The transitions that were relevant to the conformational changes of GK were addressed through trajectory analyses were generated during molecular dynamics (MD) simulations. The MD results were found to correlate best with our recent findings regarding the genetic and enzymatic characterization of R308K mutant 15.

**Figure 1:**
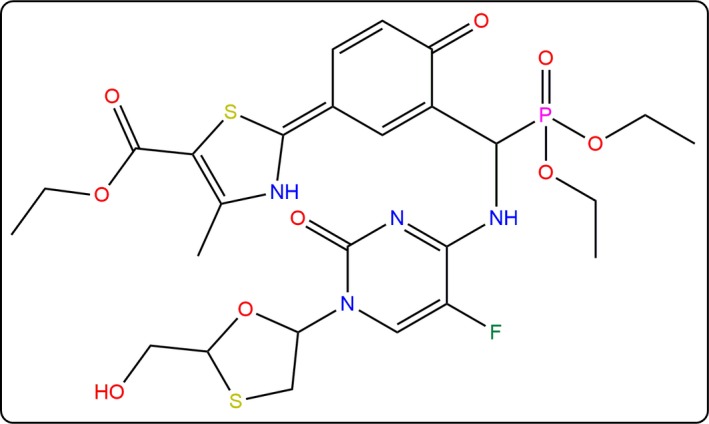
Structure of ethyl 2‐[3‐[diethoxyphosphoryl‐[[5‐fluoro‐1‐[2‐(hydroxymethyl)‐1,3‐oxathiolan‐5‐yl]‐2‐oxopyrimidin‐4‐yl]amino]methyl]‐4‐oxocyclohexa‐2,5‐dien‐1‐ylidene]‐4‐methyl‐^3^H‐1,3‐thiazole‐5‐carboxylate (YNKGKA4).

## Materials and methods

All of the *in silico* studies were carried out using molecular operating environment (moe) 16.

### Preparation and processing of glucokinase structure

The X‐ray crystal structure of GK was retrieved from the Protein Data Bank (http://www.rcsb.org/pdb/search/structidSearch.do?structureId=3F9M), with glucose in the active site and a pre‐existing activator molecule in the allosteric site 17. The structure was loaded into the moe working environment and the structure was observed to have the hetero atoms such as d‐glucose and a pre‐existing GKA 2‐amino‐4‐fluoro‐5‐[(1‐methyl‐1 h‐imidazol‐ 2‐yl)sulfanyl]‐*n*‐(1,3‐thiazol‐2‐yl)benzamide in addition to the water molecules. Such hetero atoms were removed from the crystal structure prior to processing. The structure was protonated using a generalized Born volume integral electrostatic functional form with a distance cut‐off of 10 Å and a dielectric constant of 1. Furthermore, the protonated structure was subjected to energy minimization under a Merck Molecular Force Field (MMFF94x) to an RMS gradient of 0.05 Å 18. The energy minimized structure was treated as a wild‐type GK model. The energy minimized conformation of wild‐type GK was used to construct the R308K mutant model where arginine was replaced at 308th position by a lysine residue. The mutant structure was subjected to protonation followed by energy minimization the same as that for wild‐type GK.

The refined structures of wild‐type and R308K mutant models were subjected to MD simulations individually using NPT statistical ensemble 19 and the Nosé‐Poincare‐Anderson algorithm 20. The initial temperature was set to 30 K and raised to a production phase temperature of 300 K in 100 ps with a time step of 4 fs, where the pressure was held fixed throughout the simulations with an ionic concentration of 0.145 m. The constrains were restricted to light bonds and the equations of motions made discrete with a time step of 0.002. The production phase was carried out for 20 000 ps and the position and velocities of the trajectories were saved every 0.5 ps. The low energy conformations of the wild‐type and mutant GK models obtained at the end of MD simulations were used for further investigations 21.

### Generation of GK–glucose complexes

The GK–glucose complexes were developed for the wild‐type and R308K mutants by performing individual molecular docking processes. Molecular docking was carried out using a docking module of moe where, initially, the optimized conformations obtained in MD simulations were loaded into a graphical area and the stabilized conformation of the glucose was docked into the active site regions defined by Thr168, Lys169, Asn204, Asp205, Asn231, Glue256 and Glu290 residues with *XYZ* coordinates of −26.32, −4.56 and 12.89 Å, respectively. The docking conformations were generated using a triangle matching docking placement methodology 22 and the poses were rescored using the London dG scoring function 23. Multiple conformations were generated in each docking method and the pose with the lowest score was chosen for the analysis.

The GK–glucose complexes of wild‐type and R308K mutant models were subjected to energy minimization followed by MD simulations with the same parameters that were used for the MD studies of GK structure alone. This step was performed to determine the impact of the R308K mutation on the binding efficiency and stability of the glucose molecule in the active site.

### Generation of GK–glucose–YNKGKA4 complexes

To determine the effect of YNKGKA4 on the GK–glucose complexes, another molecular docking process was carried out. In this docking process, the GK–glucose complexes of wild‐type and mutant models obtained during the first step of the molecular docking process were taken as the receptor molecules and the optimized conformation of the YNKGKA4 was treated as a ligand molecule. Individual docking processes were carried out for the wild‐type and mutant GK–glucose complexes using the same docking parameters that were used for the docking of the glucose molecule. Among the docking complexes of GK–glucose–YNKGKA4, the pose with the lowest docking score was taken for further analysis.

The role of YNKGKA4 in the super‐open and closed transition states of GK under wild‐type and R308K mutant conditions was identified by performing a further run of MD simulations. The production phase was carried out for 20 000 ps until stability was attained in the energy and RMSD levels. The trajectories were analyzed every 100 ps to allow quick observation of the transitions from the super‐open to the closed state.

## Results and Discussion

**Figure 2:**
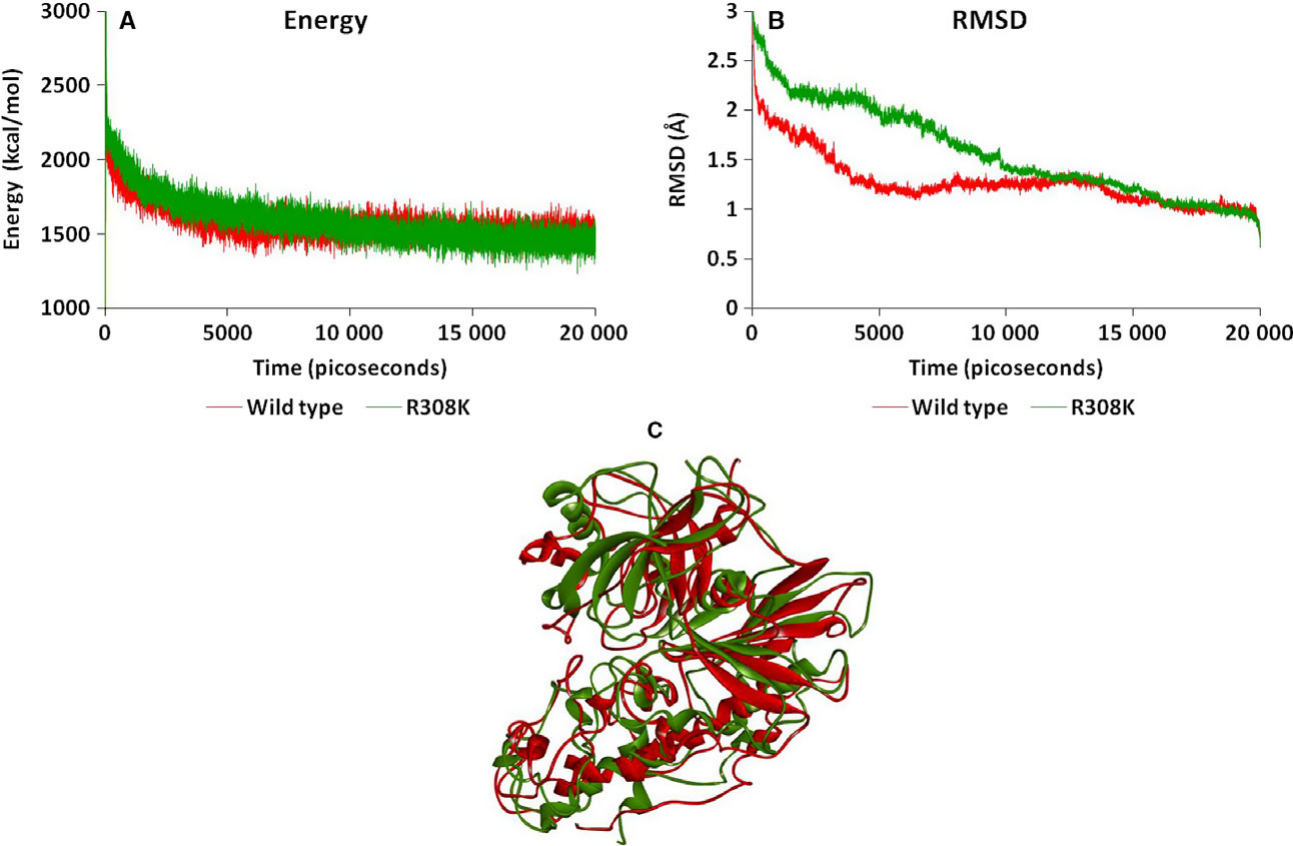
(A) Energy plot of wild‐type and R308K mutant GK models showing the energy levels during the 20 000 ps simulation period. (B) RMSD plot of wild‐type and R308K mutant GK models showing the RMSD fluctuations during the 20 000 ps simulation period. (C) Superimposed conformations of wild‐type (red) and R308K mutant (green) GK models.

The present study aimed to investigate the allosteric process of GK that is associated with the conformational transition from the super‐open to closed state, as mediated by the potential GKA molecule YNKGKA4. The investigation was started with the generation of a stabilized conformation of the GK molecule that was treated as the wild‐type and also used for generation of the R308K mutant GK model 9. The mutation could be responsible for the altered enzyme activities that were observed in our previous studies 15. The altered catalytic activity is a result of the altered conformation that occurs because of mutation. To determine the conformational variations and their functional implications, we performed a comparative investigation of the wild‐type and R308K mutant GK using molecular modelling studies. The mutant model has variable energy and RMSD levels compared to the wild‐type model. Furthermore, there were clear conformational variations in the mutant structure that could reflect the functionality of the molecules. The wild‐type model has an energy level of ~ 1600 kcal·mol^−1^, whereas this energy level was observed to be ~ 1500 kcal·mol^−1^ for the mutant GK structure. In the same way, the RMSD of wild–type GK was observed to be stabilized at ~ 0.9 Å, whereas, for mutant, it was stabilized at 0.4 Å. The superimposition of the stabilized conformations of wild‐type and mutant GK models showed an RMSD value of 5.32 Å, indicating the huge backbone conformational variations (Fig. 2). Although there was mild variation in energy and RMSD fluctuations during 20 000 ps MD simulations, the final conformations obtained at the end of production phase demonstrated huge variations that are reflected in the backbone fluctuations indicated by the RMSD of 5.32 Å. Such energy, RMSD and backbone variations explain the impact of the R308K mutation on the conformation of GK and thereby the functional variations responsible for the altered activities of GK.

**Figure 3:**
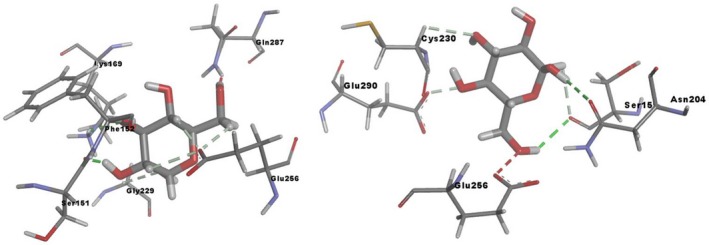
Binding mode orientation of the glucose molecule in the wild‐type (left) and R308K mutant (r) GK structures.

Such conformational variations occurring as a result of mutation will have an impact on the substrate binding affinities and orientations in the active site. This was investigated using a comparative molecular docking process where the glucose molecule was observed typically to show a different binding mode orientation in the active site of mutant GK. The docking score of wild‐type GK–glucose complex was observed to be −12.59 kcal·mol^−1^ and this score was observed to be high with the mutant GK where −10.21 kcal·mol^−1^ was observed. Furthermore, the orientation of glucose was also found to be different, in that it was observed to interact with Ser151, Phe152, Lys169, Gly229, Glu256 and Gln287 residues by means of hydrogen bonding in the wild‐type GK, whereas the glucose molecule was found to interact with Ser151, Asn204, Cyc230, Glu256 and Glu290 residues in the mutant GK (Fig. 3). There was loss in the bonding pattern in the mutant model, where three different residues such as Asn204, Cyc230 and Glu290 came into contact with glucose, compared to the wild‐type docking pattern. Such a variable bonding pattern could be responsible for the altered orientation of the glucose molecule in the binding site cavity. These variations could explain the variation in the affinity levels of the glucose molecule in the active site of mutant GK and thereby the reduced rate of glucose catalysis leading to the hyperglycemic condition.

These observations were strengthened further by running MD simulations for GK–glucose complexes of both wild‐type and mutant models. Interestingly, we observed that, during MD simulations of the wild‐type GK–glucose complex, the super‐open conformation was converted to the closed‐state during the simulation period and the glucose molecule was found to stay in a stable condition in the active site cavity. This phenomenon was different with the R308K mutant GK–glucose complex, where we observed that there is no transition of the super‐open state to the closed state and, as a result of this, the glucose molecule was observed to move out of the cavity gradually. These observations could confirm the impact of R308K mutation on the altered activity that is responsible for the T2D condition.

**Figure 4:**
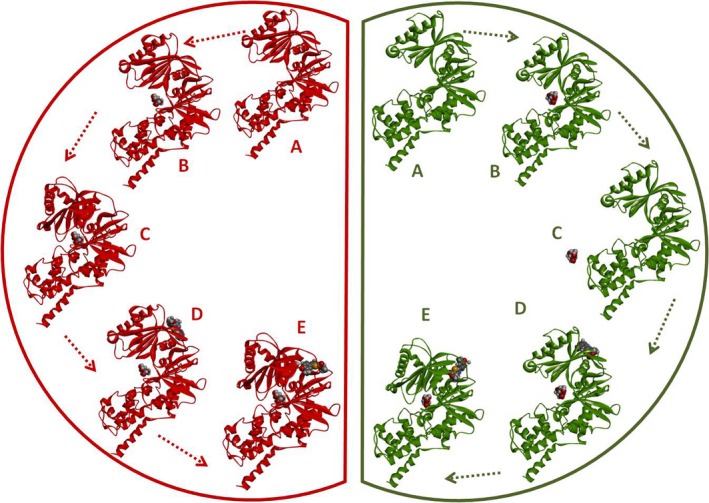
Conformational transitions of wild‐type (red) and R308K mutant (green) GK models in the presence and absence of YNKGKA4. Wild‐type GK: (A) super‐open state of GK obtained at the end of the MD simulations. (B) GK–glucose docking complex in the super‐open state. (C) Conversion of the super‐open state of GK–glucose complex into the closed state during MD simulations. (D) Molecular docking of YNKGKA4 into the super‐open state of GK–glucose complex. (E) Conversion of the super‐open state of GK–glucose complex into the closed state in the presence of YNKGKA4 during the simulation period. R308K mutant GK: (A) super‐open state of GK obtained at the end of the MD simulations. (B) GK–glucose docking complex in the super‐open state. (C) Removal of glucose molecule from the active site cavity of the super‐open state of GK during MD simulations. (D) Molecular docking of YNKGKA4 into the super‐open state of GK–glucose complex. (E) Conversion of the super‐open state of GK–glucose complex into the closed state in the presence of YNKGKA4 during the simulation period.

The next and most exciting step of our investigation was the impact of YNKGKA4 activator molecule and its role on the conformational transitions of GK–glucose complexes. The GK–glucose complexes of wild‐type and mutant models were taken in the super‐open state and the YNKGKA4 molecule was docked into the allosteric site. These GK–glucose–YNKGKA4 complexes of both wild‐type and mutants were subjected to individual MD simulations. The results obtained were interesting, in that the super‐open conformations of both of the complexes were converted into the closed state during the simulation period. Furthermore, glucose and activator molecules were found to be stable throughout the simulations. During earlier simulation phases where there was no activator molecule, glucose was expelled out of the cavity in the R308K mutant complex. However, this phenomenon did not occur when the activator molecule was in a bound condition at the allosteric site, thereby signifying its role in glucose binding to the active site (Fig. 4).

These MD simulations of GK, GK–glucose and GK–glucose–YNKGKA4 complexes explain the impact of the R308K mutation on the protein conformation and altered catalysis, as well as the potential role of YNKGKA4 activator molecule with respect to inducing transition states of GK from the super‐open state to the closed condition both in wild‐type and mutant models.

## Conclusions

In our earlier studies, we determined the activation potential of YNKGKA4 on human GK but not its actual mechanism. Hence, in the present study, we aimed to investigate its molecular mechanism, paving the way for the development of more potential GKAs. Our investigations clearly indicate the impact of the YNKGKA4 activator molecule on the super‐open and closed transition states of GK–glucose complexes. YNKGKA4 was observed to induce the closed state of GK both in wild‐type and mutant GK structures, which is the essential transition state for glucose catalysis in the binding site. The activator molecule was observed to potentially operate the transition states in the mutated condition, also thereby activating the mutant GK, which is the desirable property of the molecule. The super‐open and closed transition mechanisms were explained clearly through MD simulation studies that helped to explore the activation mechanism both under wild‐type and mutated conditions. The results observed from MD simulations both in the presence and absence of YNKGKA4 could reveal its significance at the molecular level. The *in vitro* investigations from the earlier studies confirmed the efficacy of GK activation to specific folds and suggest that the molecule is an effective therapeutic agent for use in the control and management of T2D.

## Author contributions

NKY and PKJA designed and supervised the research and prepared the manuscript. NKY, KK, RK and PKJA performed the experiments. NKY, KK, RK and PKJA analyzed the data.
